# Association between Multiple Trace Elements, Executive Function, and Cognitive Impairment with No Dementia in Older Adults

**DOI:** 10.3390/nu16071001

**Published:** 2024-03-29

**Authors:** Seung-Woo Ryoo, Baek-Yong Choi, Seok-Yoon Son, Kun-Hee Oh, Jin-Young Min, Kyoung-Bok Min

**Affiliations:** 1 Department of Preventive Medicine, College of Medicine, Seoul National University, Seoul 03080, Republic of Korea; yeon3174@snu.ac.kr (S.-W.R.);; 2 Veterans Medical Research Institute, Veterans Health Service Medical Center, Seoul 05368, Republic of Korea; 3 Institute of Health Policy and Management, Medical Research Center, Seoul National University, Seoul 03080, Republic of Korea

**Keywords:** trace element, cognitive performance, executive function, Bayesian kernel machine regression

## Abstract

Many studies suggest a significant association between individual essential trace elements (ETEs) and cognitive impairment in older adults, but evidence of the synchronized effect of multiple ETEs on cognitive function is lacking. We investigated the association between multiple ETEs, cognitive impairment with no dementia (CIND), and executive function in older Korean adults, using the Bayesian kernel machine regression (BKMR) model. Three hundred and thirty-six older adults were included as the study population and classified as the CIND and control groups. Blood manganese (Mn), copper (Cu), zinc (Zn), selenium (Se), and molybdenum (Mo) were measured as relevant ETEs. The frontal/executive tests included digit symbol coding (DSC), the Korean color word Stroop test (K-CWST), a controlled oral word association test (COWAT), and a trial-making test (TMT). Overall, the BKMR showed a negative association between multiple ETEs and the odds of CIND. Mn was designated as the most dominant element associated with the CIND (PIP = 0.6184), with a U-shaped relationship. Cu and Se levels were positively associated with the K-CWST percentiles (β = 31.78; 95% CI: 13.51, 50.06) and DSC percentiles (β = 25.10; 95% CI: 7.66, 42.53), respectively. Our results suggest that exposure to multiple ETEs may be linked to a protective mechanism against cognitive impairment in older adults.

## 1. Introduction

Aging is a prominent public health challenge facing humanity today, where the proportion of the population aged 65 or older is projected to rise from 8.5 to 16.7 percent (2015~2050) [1]. As survival rates and life expectancy among the elderly continue increasing, age-related neurodegenerative diseases have emerged as a significant healthcare problem [2,3]. In particular, Alzheimer’s disease (AD) or related dementia causes irreversible functional impairment and jeopardizes the daily lives of patients’ families. Contemporarily, there is no curative treatment available for dementia; thus, early prevention in the preclinical stage is essential [4]. Cognitive impairment with no dementia (CIND) denotes a decline in cognitive function beyond the typical age-related deterioration, yet not reaching the severity required for a dementia diagnosis [5]. Besides the increased risk of developing dementia, patients with CIND experience higher rates of disability or mortality, highlighting the importance of identifying risk factors in this population [6,7].

Essential trace elements (ETEs) are imperative nutrients linked to cognitive impairment. ETEs are constituents of normal human physiology, of which both deficiency and excess can induce toxic effects [8]. For instance, overflowing accumulation and minimal absorption of copper (Cu) due to a transporter mutation cause severe genetic syndromes [9]. Despite the small recommended daily intake (<100 mg), ETEs are indispensable as enzyme cofactors or structural stabilizers of the large molecule [10]. Prior studies have observed that multiple ETEs influence the neuropathological process. A partial deficiency in a manganese (Mn)-dependent antioxidant enzyme increased brain amyloid-beta (Aβ) levels and Aβ plaque burden in vivo, which is an underlying mechanism of dementia [11]. Likewise, AD model mice with downgraded copper/zinc (Zn) superoxide dismutase (SOD1) showed accelerated Aβ synthesis and memory impairment [12]. Among the AD model mice, a higher level of cortex DNA damage was found in the Se-deficient diet group compared to the control [13]. Molybdenum (Mo) exposure to murine microglial cells triggered reactive oxygen species generation and pyroptosis [14]. Epidemiological evidence has been accumulated regarding the relationship between each ETE and cognitive abilities. For example, Tong et al. (2014) reported that low blood Mn levels were correlated with cognitive decline and clinical dementia scores in Chinese older adults [15]. An Italian cohort found that blood Se in CIND and AD patients was lower than in the control group [16]. A longitudinal study identified the role of Se, reporting a reduction in cognitive scores with increasing fingernail Se in the Portuguese elderly [17]. A dietary survey of a large Chinese population observed that intake of Cu was negatively associated with cognitive function, while Zn and Mn showed no significance [18]. However, findings on the relationship between ETE and cognitive function are inconsistent, and this may arise from variations in ETE exposure among samples due to a lack of consideration for co-exposure to multiple ETEs.

Various statistical methods have been developed to examine the relationship between multiple exposures and public health. Dimensional reduction methods, including principal component analysis or observation grouping methods such as classification and regression trees, have been utilized, yet several challenges in estimating multiple exposure effects remain unresolved [19]. Recently, a new approach for estimating the health effects of multiple exposures, machine learning-based Bayesian kernel regression (BKMR), has been introduced [20]. This method uncovers non-linear relationships across multiple exposure components and analyzes integrated independent variables simultaneously [20]. Similarly, it can model the interaction effects between exposures and address the multicollinearity of mixture components by hierarchical variable selection [20]. Bobb et al. performed a simulation analysis and successfully demonstrated the robustness of the statistical method in predicting the exposure mixture–health outcome function [20]. BKMR has been applied to dementia research, yet scarce evidence on its association with ETEs exists, with most studies focusing on single screening tests [21,22,23,24]. This study examined the associations between multiple ETEs and cognitive function. Cognitive function was assessed based on the presence of CIND and executive performance. Impaired executive functions such as inhibition, planning, or decision-making precluded patients’ independence in their daily activities [25,26]. Impaired executive functions were known to be an early predictor of progression from MCI to dementia [27]. We conducted a BKMR analysis to investigate the associations between multiple ETEs, CIND, and executive function in older Korean adults.

## 2. Materials and Methods

### 2.1. Study Population

The Korea Dementia Initiative Veteran’s Affairs (KODIVA) is an ongoing study of patients who visited the Department of Neurology at the Veterans Health Service Medical Center in Seoul, South Korea. During 2021–2022, 600 veterans aged 60 years and older were voluntarily enrolled via convenience sampling and participated in clinical examination, neuropsychological evaluation, and face-to-face questionnaire surveys. The inclusion criteria were as follows: (1) those with a subjective sense or experience of cognitive decline, (2) those able to receive clinical tests and answer survey questionnaires, and (3) those who could provide informed consent to participate in the study. The participants were excluded if board-certified neurologists diagnosed the following sets of diseases: (1) dementia (International Classification of Disease, 10th Revision, code F00–F09, and G30); (2) brain infarction, cerebral hemorrhage, or Parkinson’s disease; and (3) severe medical conditions (e.g., cancer, intellectual disability, or psychiatric illnesses).

Clinical assessment was designed to confirm the presence or absence of cognitive impairment and provide a diagnosis of Alzheimer’s and dementia. Clinical examination included a clinical interview, neuropsychological evaluation, standard laboratory testing, and brain imaging, which physicians, neuropsychologists, and nurses conducted. Physicians performed a clinical interview of general medical, neurological, and family history; neurological signs (i.e., focal signs and Parkinsonism); and laboratory testing of vitamin B12, thyroid-stimulating hormone, and cholesterol levels. They also analyzed a CT or MRI scan of the brain of electronic medical records when evaluating patients with suspected dementia. Neuropsychologists assessed cognitive function with a neuropsychological test battery and daily functioning with instrumental activities of daily living. Nurses collected participants’ data on socio-demographic information, lifestyle, and personal factors. The clinical staff diagnosed CIND, referring to all available data from clinical examinations and neuropsychological evaluations [28]. Individuals with CIND are defined as those without dementia and with cognitive impairment.

This study conducted a cross-sectional analysis of blood ETEs, CIND, and executive function. Among 600 study participants, 336 who were tested for blood ETEs were eligible participants for the current study. Of them, 199 participants were designated as those with CIND, and 137 were those with normal cognition.

### 2.2. Sample Collection and Measurement of Blood ETEs

Whole blood or serum samples were collected from all participants and measured by an inductively coupled plasma mass spectrometer, Agilent ICP-MS 7900 (Agilent Technologies, Tokyo, Japan), equipped with standard Ni sampling and skimmer cones and an Ultra High Matrix Introduction system. Calibration standards were prepared from an Agilent multi-element environmental calibration standard (p/n 5183-4688) containing 10 ppm each of Cu, Mn, Mo, Se, and Zn. The internal standard (Sc, Ge, Rh, In, and Bi) for the quantitative analysis was made in 1% HNO_3_ and 0.5% HCl for the diluted sample. Triton C-X-100, Butanol, EDTA, ammonium hydroxide, and water were added to the alkaline solution. Dilution and sample preparation were performed under a clean hood to prevent contamination by atmospheric particulates. An amount of 100 μL of the standard was added to a 15 mL polypropylene tube, and then 22 mL of diluent was added with a dispenser. Next, 100 μL of samples and the control were dispersed in the dispensed diluent. Diluted samples were vortex-mixed and analyzed with Agilent ICP-MS.

### 2.3. Assessment of Frontal/Executive Function

Frontal/executive function was assessed by the Seoul Neuropsychological Screening Battery (SNSB)-Core (SNSB-C). The SNSB is a representative comprehensive neuropsychological evaluation tool in Korea and is widely used by dementia experts [29]. SNSB-C has proven to be an effective replacement for the original version, showing comparable sensitivity, specificity, and positive predictive value with a shorter test time (around 40 min) [30]. The frontal/executive domain is assessed by four tests: digit symbol coding (DSC), the Korean color word Stroop test: 60 s (K-CWST: 60 s), the controlled oral word association test: animal + ‘ㄱ’ (COWAT: animal + ㄱ), and the trail-making test—elderly: part b (TMT-E: B). Each test score was as a standardized percentile, stratified by age, sex, and education level.

### 2.4. Covariates

We measured demographic properties, health behaviors, and medical history as covariates included in the analyses. Demographics included age (60–64, 65–69, 70–74, 75–79, ≥80 years), sex (male, female), and education level (below middle school, middle or high school, college). Health behaviors included smoking status (current or not) and alcohol consumption (within the past year or not). Medical history of hypertension (yes, no), diabetes (yes, no), and dyslipidemia (yes, no) were obtained by self-report during the previous diagnosis.

### 2.5. Statistical Analysis

A descriptive analysis was conducted to show the distribution of the participants’ general characteristics. The difference in the prevalence of variables was identified using the Chi-squared or Fisher’s exact test. Concentrations of blood ETEs were natural log (ln)-transformed to satisfy a normal distribution. General linear and multivariable regression analyses investigated the association between blood ETEs, CIND, and executive function. The categorical variable for the status of ‘CIND’ and the continuous percentile value of each executive test were used as dependent variables. First, single-element regression models with one ETE as an independent variable were applied. Second, multi-element regression models were involved with all five ETEs as independent variables. Both single- and multi-element regression models were adjusted for age, sex, and education level, as previous studies have identified three confounding factors strongly associated with the development of mild cognitive impairment [31,32].

The BKMR model was constructed to explore the joint associations of the ETE mixture with cognitive scores. To analyze the binary outcome (odds for being CIND), a Probit extension of the Bayesian kernel machine regression (BKMR-P) model was additionally built. BKMR is a novel non-parametric method developed to estimate multiple exposure–response functions and investigates these exposures’ cumulative, non-linear, or interactive effects [20,33]. By employing a kernel function, this method flexibly estimates the multivariable exposure–response relationship, accommodating non-linear and non-additive effects, with potential confounding factors adjusted [33]. To tackle the multicollinearity issue, hierarchal variable selection was incorporated. The model initially categorizes highly correlated exposures into groups and subsequently performs variable selection on the groups of correlated exposures and the individual exposures within each group concurrently [33].

After the model fitting, posterior inclusion probabilities (PIPs) ranging from 0 to 1 were calculated to assess the contribution of each ETE to the BKMR models. The overall impact of the exposure set was measured by comparing the difference in cognitive outcomes when all of the ETEs were placed at a specific quantile, compared with the median. The individual effect of each exposure component was estimated by calculating the difference in cognitive outcomes when one ETE concentration varied from the 25th percentile to the 75th percentile under all the other ETE concentrations placed at their 25th, 50th, and 75th percentile. In addition, univariate exposure–outcome functions were visualized when all the other ETEs were fixed at the median to investigate the dose–response or non-linear relationships between each exposure and cognitive outcome. Lastly, the bivariate exposure–outcome functions for one varying ETE on cognitive outcomes at different quantiles of the second ETE concentration (10th, 50th, and 90th percentiles) while the other ETEs were fixed at their median were visualized to observe the interactive effects of ETE pairs.

To confirm the robustness of these findings, sensitivity analyses were conducted after excluding subpopulations of the sample. Among the 199 participants diagnosed with CIND, medical experts further identified 13 participants at a high risk of dementia based on clinical examination. The conventional regression analyses were performed as sensitivity analyses on the sample without high-risk participants.

The statistical analyses were conducted with SAS 9.4 software (SAS Institute, Cary, NC, USA), and the BKMR analysis was performed in R software (version 4.3.2; R Development Core Team) using the ‘bkmr’ package [33]. Statistical significance set to a *p*-value threshold of 0.05 was used.

## 3. Results

The general characteristics of 336 elderly populations are displayed in Table 1. The distribution of the covariates was slightly different between the CIND group and the control group but not statistically significant. The mean age (74.93) and the proportion of women (56.78%) were higher in the CIND group. The control group showed a lower degree of education, with a lower proportion of college-educated participants (24.09%). The percentage of current smokers was higher in the CIND group (4.52%), though most participants have quit or never smoked. The CIND group had a higher prevalence of recent alcohol consumption (37.19%), hypertension (56.28%), and diabetes (26.13%), but not of dyslipidemia (45.73%).

Table 2 shows the results of the multivariable logistic regression analysis with the status of CIND as the dichotomous outcome. The odds ratio for Zn was significant in the single-element model within a 95% confidence interval (OR = 0.26, 95% CI: 0.07 to 0.99). However, no exposure variable was found significant in the multiple-elements model.

Coefficients from the general linear regression analysis are collected in Table 3. Se was positively associated with DSC score percentiles in both the single-element (β = 25.57, 95% CI: 8.69 to 42.45) and multiple-element models (β = 25.10, 95% CI: 7.66 to 42.53). Of the other ETEs, only Cu had positive associations with K-CWST: 60 s score percentiles in both single-element (β = 30.90, 95% CI: 13.19 to 48.62) and multiple-element models (β = 31.78, 95% CI: 13.51 to 50.06).

The contribution of each ETE in an individual BKMR model is quantitatively presented as a PIP in Table 4. Regarding the odds of CIND, Mn was identified as the most contributing variable (PIP = 0.6184), followed by Zn (PIP = 0.3756). Consistent with the conventional regression analyses, Se was the dominant variable for the DSC percentiles (PIP = 0.9982), while Cu was the largest contributor for K-CWST: 60 s percentiles (PIP = 0.9054).

**Figure 1:**
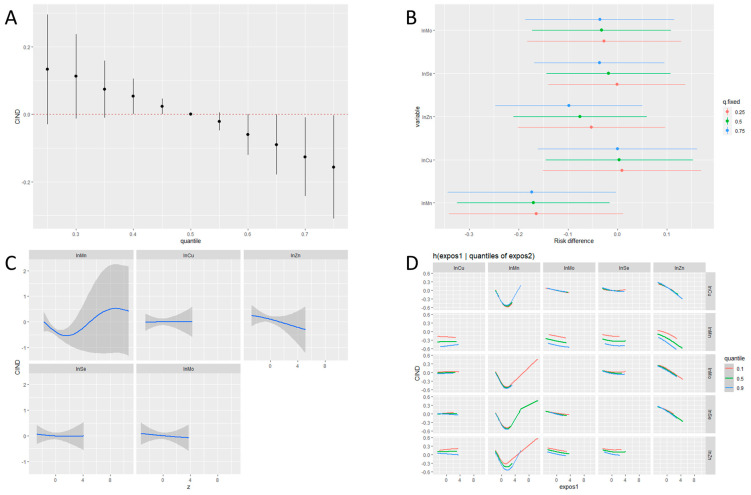
Joint associations between ETEs and CIND using BKMR. The model was adjusted for age, sex, and education. (**A**) Joint association of the ETEs mixture and 95% CIs on CNID risk when all ETEs at particular percentiles were compared to all ETEs at their 50th percentile. (**B**) Univariate exposure effect on CIND with 95% CIs defined as the response difference when each ETE changes from its 25th to 75th percentiles where the remaining ETEs are fixed at a particular level (25th, 50th, and 75th percentiles). (**C**) Univariate exposure−response functions and 95% CIs for individual ETEs, when other ETEs were fixed at their medians. (**D**) Bivariate exposure−response functions for each ETE when the second ETE was fixed at a different level (10th, 50th, and 90th percentiles) and the remaining ETEs were fixed at their medians.

Figure 1 shows the collective results of the BKMR-P analysis. The odds of CIND were negatively associated with the increasing concentration of ETEs in the exposure set (Figure 1A). The odds of CIND significantly decreased as the Mn concentration varied from the 25th to the 75th percentile when the concentration percentiles of other ETEs were fixed at the median or 75th percentile (Figure 1B). The univariate exposure–response analysis suggested a U-shaped relationship between blood Mn and CIND risk (Figure 1C). Visual inspection of the bivariate exposure–response functions indicated that as the Mn quantile increased, the intercepts of other elements decreased, with the slopes remaining relatively consistent (Figure 1D). This alteration suggests Mn only had an additive interaction with other ETEs.

Sensitivity analyses after excluding participants at high risk for dementia produced findings similar to those of the main analyses. No significant association was observed between blood ETEs and CIND. In contrast, blood Cu and Se were positively associated with K-CWST: 60 s (β = 33.26; 95% CI: 14.88 to 51.64) and DSC score percentiles (β = 24.64; 95% CI: 6.68 to 42.60), respectively, in the multiple-element model (Tables S1 and S2).

## 4. Discussion

This study investigated the association between multiple ETEs, CIND, and executive function in older adults using the BKMR model. The BKMR-P showed a negative association between the ETE mixture and CIND risk, and Mn contributed to the largest portion of the model. Additionally, Mn exhibited a non-linear relationship with the outcome variable. The conventional regression showed the dominant role of Cu and Se in altering the specific domains of executive function.

As far as we know, no study identifies the potential link between multi-element exposure and executive function. Some studies have suggested a positive association of combined multi-element exposure with better cognitive function [23,24,34]. A recent BKMR study conducted on 2181 Chinese elderly participants analyzed the joint association between blood ETE mixture (Mn, Se, Cu, chromium) and cognition function assessed by the Mini-Mental State Examination (MMSE) [23]. They found that the ETE mixture was positively associated with MMSE scores, and Se (posterior inclusion probabilities, PIPs = 0.915) was the most important contributor within the ETE mixture. Cheng et al. (2022) explored an association between multiple urine ETEs and MMSE in 3815 adults aged over 60 [24]. From the analysis of BKMR, higher urinary concentrations of ETEs were related to MMSE scores in a dose–response manner. Se levels showed the highest PIP (0.56) among the five elements (Se, Mo, vanadium, cobalt, strontium). Duan et al. (2023) applied minor absolute shrinkage and selection operator regression to investigate the effect of multi-element combined exposure (Se, Cu, Zn, iron, lead, calcium) on cognitive function in 416 elderly participants [34]. Log-transformed levels of Se (β = 0.32; *p* = 0.007) and Cu (β = 0.75; *p* = 0.048) were positively associated with a composite z score from nine cognitive tests.

Our findings showed a downward trend of cognitive impairment odds as the mixed ETE concentration increased through the visual exploration of BKMR-P. We found that Mn in the multi-exposure model was the most significant contributor to CIND, showing a U-shaped relationship between them. Previous studies have shown a considerable effect of Mn on cognitive impairment [35,36]. Larvie et al. (2022) analyzed the 2013–2013 NHANES data and found that the digit symbol substitution test score was inversely associated with blood Mn among the subjects in the highest quartile (*p* = 0.003) [35]. A meta-analysis of 17 epidemiologic studies found a significant decrease in serum Mn concentration in the cognitive impairment group (AD or MCI patients) compared to healthy controls (standardized mean difference: −0.37; 95% CI: −0.6 to −0.13) [36]. Contrarily, excess exposure to Mn was also a risk factor for neurodegeneration, thus explaining the U-shaped relationship found in our results [35].

While this is the first study to report the importance of Mn in cognitive impairment through the estimation of multiple elementary exposures in BKMR, our finding is plausible given the biological reactions of Mn in mediating the physiological and toxic actions of neuronal degeneration. Mn is an essential metallic element for human physiology, in which Mn mediates proper neuronal function via its role as a cofactor in myriad enzymatic processes [37]. Mn-dependent superoxide dismutase/superoxide dismutase 2 (MnSOD/SOD2) diminishes neuronal oxidative stress by catalyzing the degradation of superoxide anion radicals, balancing neuronal apoptosis and neurodegeneration [38,39]. Depletion of arginine by Arginase1, another Mn-dependent enzyme, inhibits the synthesis of nitric oxide and assists neuronal survival [40]. Ironically, excessive Mn accumulation impedes MnSOD activity, while high levels of Mn could also induce Aβ-related neurotoxicity in vitro and in vivo [15,41].

Regarding executive function and ETEs, the contribution of Cu and Se was particularly noteworthy. Some studies have suggested significant associations of cognitive function with Cu or Se in a single exposure model [42,43,44,45,46]. Wang et al. found a positive association between Cu intake and executive function test scores [42]. On the contrary, Meramat et al. revealed that the copper level in the toenail is associated with cognitive impairment determined by the Montreal Cognitive Assessment score (OR = 1.275; 95% CI: 1.047 to 1.552) [43]. Cu is a necessary micro-element and plays essential roles in many biochemical processes by involving neuronal metabolism and moderate physiologic reactions at the active site of multiple enzymes [47]. The reduction potency of the Cu^2+^/Cu^+^ complex provides cuproenzymes that can oxidize their substrates, notably superoxide dismutase 1, converting superoxide into dioxygen and hydrogen peroxide [48]. Furthermore, Cu-dependent enzymes partake in neurotransmitter or neuropeptide synthesis [47,49].

Se had a significant relationship with executive function in our population. A Chinese elderly study showed a dose–response relationship between nail Se level and executive test (IU Token test) score (*p* < 0.001) [44]. A US study reported an inverted-U-shaped association between serum Se and digit symbol substitute test scores [45]. However, a cross-sectional study of Australian older adults observed no association between plasma Se and self-reported executive function score (BRIEF-A) (β = −0.015; 95% CI: −0.041 to −0.011) [46]. In the biological process, Se participates in the redox mechanism in the form of selenocysteine and is incorporated into the active site of selenoproteins such as glutathione peroxidases (GPX) and thioredoxin reductases (TXNRD). GPX is acknowledged as the primary enzyme that counteracts ROS and reduces neuronal damage from oxidative stress [50]. Though biological evidence explaining the reversed association of cognitive performance with high levels of Se is scarce, previous studies have proposed the threshold range of protective Se and the possible toxicity of Se [51,52].

We investigated the effect of multiple ETEs on cognitive function in a large elderly population, particularly using a comprehensive neuropsychological assessment tool with high diagnostic validity. Our results are more clinically applicable than those drawn from analyses with simple screening methods (e.g., MMSE). Our study may serve as a rationale to persuade clinicians and health authorities to plan appropriate interventions. Our study has several limitations to consider. First, it was a cross-sectional study, and reverse causation may exist. For instance, people with high cognitive function might be able to learn health information better and thus adhere to nutritional supplements more. Further analysis based on prospective follow-up and repeated measurement samples may yield different results. Second, since the study was based on visitors within a single tertiary care center, selection bias could have distorted the result and weakened the external validity. Our sample harbors a higher percentage of retired veterans, which may have increased the baseline risk of dementia [53]. Third, residual confounding effects from covariates may not be adjusted in the analyses. Indeed, to minimize the exclusion of participants due to missing data, a few considerable variables, such as physical activity or income, were not involved. Fourth, our study does not include experimental findings to support the pathways by which ETEs induce cognitive impairment. Re-collection of blood samples and the measurement of pathologic changes (e.g., oxidative stress, DNA damage) at future follow-ups would shed light on the biological mechanism linking ETE exposure and CIND. Finally, although we have selected five exposures pertinent to cognitive function from a systematic article review, other ETEs observed to influence human cognition (e.g., chromium) were not included [54].

## 5. Conclusions

In conclusion, Mn was identified as the foremost element regarding the protective mechanism of ETEs in preventing clinically declined cognition, while Se and Cu were positively associated with executive function. However, the U-shaped association of Mn with CIND is indicative of the potential toxicity of extremely high exposure to ETEs in cognitive health. Our study implies that proper levels of ETEs within the body’s metabolism may contribute to preventing cognitive decline in the elderly population. Therefore, the public health bureau should prioritize monitoring ETE exposure to improve dementia-related interventions. Future research should focus on establishing the optimal level of ETEs to maintain cognitive function. Moreover, prospective cohort studies, including additional ETEs or other toxicants as exposure, will be needed to clarify the causal relationship between multiple ETEs and cognitive function.

## Figures and Tables

**Table 1:** General characteristics and medical conditions of the participants.

Characteristics		CIND		Normal Control		*p*-Value
		(*n* = 199)		(*n* = 137)		
Age						
60–64		14	(7.04)	6	(4.38)	0.5561
65–69		16	(8.04)	16	(11.68)	
70–74		56	(28.14)	40	(29.20)	
75–79		79	(39.70)	57	(41.61)	
≥80		34	(17.09)	18	(13.14)	
Gender						
Male		86	(43.22)	67	(48.91)	0.3035
Female		113	(56.78)	70	(51.09)	
Education						
Below middle school		55	(27.64)	46	(33.58)	0.2377
Middle or high school		80	(40.20)	58	(42.34)	
College		64	(32.16)	33	(24.09)	
Smoking (current)						
	No	190	(95.48)	133	(97.08)	0.4541
	Yes	9	(4.52)	4	(2.92)	
Drinking (within the past year)						
	No	125	(62.81)	87	(63.50)	0.8976
	Yes	74	(37.19)	50	(36.50)	
Hypertension						
	No	87	(43.72)	71	(51.82)	0.1435
	Yes	112	(56.28)	66	(48.18)	
Diabetes						
	No	147	(73.87)	105	(76.64)	0.5640
	Yes	52	(26.13)	32	(23.36)	
Dyslipidemia						
	No	108	(54.27)	69	(50.36)	0.4810
	Yes	91	(45.73)	68	(49.64)	

Values are presented as numbers (%).

**Table 2:** Association between blood ETEs (natural log-transformed) and CIND based on multivariable logistic regression.

ETEs	Single Element ^a^	Multiple Elements ^a^
	OR (95% CI)	OR (95% CI)
Mn	0.62 (0.26, 1.48)	0.72 (0.30, 1.73)
Cu	0.84 (0.25, 2.86)	1.05 (0.29, 3.80)
Zn	0.26 (0.07, 0.99)	0.29 (0.07, 1.18)
Se	0.55 (0.17, 1.82)	0.73 (0.21, 2.52)
Mo	0.90 (0.58, 1.42)	0.84 (0.53, 1.33)

OR, odds ratio; CI, confidence interval. ^a^ adjusted to age, sex, and education level.

**Table 3:** Association between blood ETEs and executive function tests based on general linear regression.

ETEs	DSC	K−CWST: 60 s	COWAT (ㄱ)	COWAT (Animal + ㄱ)	TMT−E: B ^b^
	β (95% CI)	β (95% CI)	β (95% CI)	β (95% CI)	β (95% CI)
Single element ^a^					
Mn	0.49 (−11.65, 12.63)	−0.66 (−13.05, 11.72)	−3.57 (−15.81, 8.67)	2.98 (−8.95, 14.90)	3.45 (−6.39, 13.28)
Cu	15.82 (−1.78, 33.41)	30.90 (13.19, 48.62) ^†^	9.04 (−8.76, 26.84)	2.82 (−14.54, 20.19)	1.02 (−13.27, 15.30)
Zn	10.45 (−8.34, 29.24)	11.53 (−7.63, 30.69)	−11.84 (−30.78, 7.11)	−9.30 (−27.78, 9.17)	5.72 (−9.34, 20.78)
Se	25.57 (8.69, 42.45) ^†^	12.47 (−4.92, 29.86)	6.17 (−11.07, 23.41)	3.95 (−12.85, 20.75)	8.93 (−4.89, 22.74)
Mo	−4.73 (−11.14, 1.67)	−1.12 (−7.67, 5.43)	0.12 (−6.36, 6.60)	0.61 (−5.70, 6.92)	2.48 (−2.69, 7.65)
Multiple elements ^a^					
Mn	−4.02 (−16.41, 8.37)	−6.12 (−18.73, 6.50)	−3.98 (−16.65, 8.68)	3.62 (−8.76, 16.01)	2.60 (−7.58, 12.78)
Cu	15.49 (−2.46, 33.45)	31.78 (13.51, 50.06) ^†^	12.36 (−5.99, 30.71)	3.87 (−14.07, 21.81)	0.44 (−14.32, 15.20)
Zn	1.09 (−18.59, 20.77)	4.83 (−15.20, 24.87)	−15.37 (−35.48, 4.74)	−12.45 (−32.12, 7.21)	3.93 (−12.06, 19.92)
Se	25.10 (7.66, 42.53) ^†^	12.25 (−5.50, 30.00)	10.00 (−7.82, 27.83)	6.12 (−11.31, 23.54)	8.10 (−6.19, 22.39)
Mo	−3.48 (−9.91, 2.94)	0.44 (−6.10, 6.98)	0.04 (−6.52, 6.61)	0.50 (−5.92, 6.92)	2.93 (−2.33, 8.19)

β, coefficient from linear regression; CI, confidence interval, ^†^ *p* < 0.01. ^a^ adjusted to age, sex, and education level. ^b^ analyzed except for 5 participants who were unable to perform the test.

**Table 4:** Posterior inclusion probabilities of essential trace elements in each BKMR model.

Cognitive Function	Essential Trace Elements				
	Mn	Cu	Zn	Se	Mo
CIND	0.6184	0.2388	0.3756	0.2874	0.2296
Executive function					
DSC	0.0180	0.2360	0.0406	0.9982	0.0402
K-CWST: 60 s	0.0812	0.9054	0.0642	0.2808	0.0420
COWAT (ㄱ)	0.0910	0.1208	0.0830	0.3766	0.0434
COWAT (animal + ㄱ)	0.0744	0.0414	0.0808	0.2834	0.0310
TMT-E: B	0.0194	0.0348	0.0334	0.1558	0.0670

## Data Availability

The data that support the findings of this study are available from the corresponding author upon reasonable request. The data are not publicly available due to privacy.

## Supplementary Materials

The following supporting information can be downloaded at: https://www.mdpi.com/article/10.3390/nu16071001/s1, Table S1: Association between blood ETEs (natural log-transformed) and CIND by multivariable logistic regression from the sensitivity analysis; Table S2: Association between blood ETEs and executive function tests by general linear regression from the sensitivity analysis.
