# Supplementary material for: Association between Multiple Trace Elements, Executive Function, and Cognitive Impairment with No Dementia in Older Adults
Source: Nutrients. 2024 Mar 29;16(7):1001. doi: 10.3390/nu16071001 (PMC11013674; doi:10.3390/nu16071001)
Supplement: Supplementary file 1 [file nutrients-16-01001-s001.zip › nutrients-2904294-supplementary.docx]

| **Table S1**. Association between blood ETEs (natural log-transformed) and CIND by multivariable logistic regression from the sensitivity analysis | | |
| --- | --- | --- |
| **ETEs** | **Single element^a^** | **Multiple elements^a^** |
|  | **OR (95% CI)** | **OR (95% CI)** |
| Mn | 0.57 (0.23, 1.43) | 0.66 (0.27, 1.63) |
| Cu | 0.79 (0.23, 2.75) | 1.00 (0.27, 3.67) |
| Zn | 0.30 (0.08, 1.14) | 0.33 (0.08, 1.36) |
| Se | 0.64 (0.18, 2.24) | 0.86 (0.24, 3.16) |
| Mo | 0.92 (0.59, 1.46) | 0.86 (0.54, 1.37) |
| OR, odds ratio; CI, confidence interval,  ^a^ adjusted to age, sex, and education level. | | |

| **Table S2**. Association between blood ETEs and executive function tests by general linear regression from the sensitivity analysis | | | | | |
| --- | --- | --- | --- | --- | --- |
| **ETEs** | **DSC** | **K-CWST: 60sec** | **COWAT (ㄱ)** | **COWAT (animal+ㄱ)** | **TMT-E: B^b^** |
|  | **β (95% CI)** | **β (95% CI)** | **β (95% CI)** | **β (95% CI)** | **β (95% CI)** |
| Single element^a^ |  |  |  |  |  |
| Mn | 2.20 (-9.97 , 14.37) | -0.45 (-13.00 , 12.09) | -2.81 (-15.29 , 9.66) | 4.05 (-7.97 , 16.06) | 6.11 (-2.95 , 15.17) |
| Cu | 16.95 (-0.55 , 34.45) | 32.13 (14.34 , 49.92)^†^ | 10.59 (-7.41 , 28.59) | 4.09 (-13.29 , 21.47) | 1.81 (-11.36 , 14.98) |
| Zn | 5.83 (-12.78 , 24.43) | 8.38 (-10.78 , 27.55) | -15.08 (-34.09 , 3.93) | -12.91 (-31.24 , 5.43) | 2.48 (-11.31 , 16.28) |
| Se | 24.41 (7.01 , 41.81)^†^ | 10.41 (-7.70 , 28.52) | 5.33 (-12.71 , 23.36) | 1.37 (-16.02 , 18.76) | 5.61 (-7.58 , 18.80) |
| Mo | -4.05 (-10.43 , 2.34) | -0.41 (-7.01 , 6.19) | 0.85 (-5.71 , 7.41) | 1.46 (-4.86 , 7.78) | 1.58 (-3.21 , 6.37) |
| Multiple elements^a^ |  |  |  |  |  |
| Mn | -2.04 (-14.49 , 10.40) | -5.59 (-18.37 , 7.20) | -3.03 (-15.91 , 9.86) | 5.14 (-7.32 , 17.61) | 5.80 (-3.62 , 15.22) |
| Cu | 16.40 (-1.49 , 34.30) | 33.26 (14.88 , 51.64)^†^ | 14.24 (-4.29 , 32.77) | 5.53 (-12.39 , 23.45) | 0.85 (-12.74 , 14.44) |
| Zn | -3.61 (-23.05 , 15.83) | 2.08 (-17.88 , 22.04) | -18.80 (-38.91 , 1.32) | -15.83 (-35.29 , 3.63) | 0.23 (-14.39 , 14.85) |
| Se | 24.64 (6.68 , 42.60)^†^ | 10.28 (-8.16 , 28.72) | 9.70 (-8.89 , 28.29) | 3.85 (-14.13 , 21.83) | 4.56 (-9.08 , 18.21) |
| Mo | -3.10 (-9.51 , 3.31) | 1.04 (-5.54 , 7.62) | 0.67 (-5.96 , 7.31) | 1.24 (-5.18 , 7.66) | 1.90 (-2.97 , 6.76) |
| β, coefficient from linear regression; CI, confidence interval, †P<0.01. ^a^ adjusted to age, sex, and education level. ^b^ analyzed except for 3 participants who were unable to perform the test. | | | | | |
